# Experimental Testing of Host Range of the Parasitoid Wasp *Trichogramma dendrolimi* Under Laboratory Conditions

**DOI:** 10.3390/insects16111114

**Published:** 2025-10-31

**Authors:** Aleksander A. Ageev, Anna N. Golovina, Alsu M. Utkuzova, Anastasia V. Shestopalova, Yuri S. Tokarev

**Affiliations:** 1 Center of Forest Pyrology, All-Russia Research Institute of Silviculture and Mechanization of Forestry, Krupskoy 42, Krasnoyarsk 660062, Russia; ageev_aa@mail.ru (A.A.A.); annang@list.ru (A.N.G.); anastasiya.shestopalova.01@mail.ru (A.V.S.); 2 All-Russian Institute of Plant Protection, Podbelskogo 3, Pushkin, St. Petersburg 196608, Russia; alsuvizr@mail.ru

**Keywords:** egg parasitism, laboratory culture, classical biocontrol, Siberan silk moth

## Abstract

**Simple Summary:**

*Trichogramma* is an important biocontrol agent against agricultural and forest pests. For efficient exploitation for pest control, it is important to know the host range of egg parasitoids. We isolated *T. dendrolimi* from eggs of the Siberian silk moth *Dendrolimus sibiricus.* We tested the eggs of 17 lepidopteran insect species. Successful parasitism was observed on eggs of 14 species, including the original host. The highest yield of the parasitoid was observed on eggs of *D. sibirucus* (33 adults/egg), the pine-tree lappet *D. pini* (32 adults/egg), the fox moth *Macrothylacia rubi* (27 adults/egg), and the tobacco hornworm *Manduca sexta* (26 adults/eggs), while the other species showed lower values by an order of magnitude, including the rusty tussock moth, the cabbage moth, the greater wax moth, and the grain moth. Up to 30 generations were reared on *D. sibiricus*, *M. sexta*, *M. brassicae*, and *S. cerealella* eggs. The parasitoid remained viable and effective against diverse lepidopteran hosts. This study broadens the known host range of *T. dendrolimi*, with some host species supporting high reproduction suitable for mass propagation.

**Abstract:**

*Trichogramma* is an important genus of egg parasitoids, applied against agricultural and forest lepidopteran pests. Known species differ in host specificity, which affects both their efficiency in field and suitability for mass rearing. In 2022, a novel strain of *T. dendrolimi* was recovered from eggs of the Siberian silk moth *Dendrolimus sibiricus* in Eastern Siberia. Freshly laid eggs of lepidopteran insects belonging to 17 species were exposed to adult *T. dendrolimi.* Besides the original host, successful reproduction was observed in the eggs of 13 species belonging to the families of Sphingidae (3 species), Noctuidae (2 species), Pyralidae (1 species), Crambidae (1 species), Erebidae (3 species), Gelechiidae (1 species), Geometridae (2 species), Nolidae (1 species), and Lasiocampidae (3 species). The maximum parasitoid yield of 33 adults per egg was observed in *D. sibiricus*, followed by *Dendrolimus pini* (32 adults/egg), *Macrothylacia rubi* (27 adults/egg), *Manduca sexta* (26 adults/egg), *Orgyia antiqua* (4 adults/egg), *Pseudoips prasinana* (3 adults/egg), *Mamestra brassicae*, *Angerona prunaria*, and *Chrysorithrum flavomaculata* (2 adults/egg), as well as *Hydraecia micacea*, *Ostrinia nubilalis*, *Galleria mellonella*, and *Sitotroga cerealella* (1 adult/egg). Thirty generations were successfully reproduced in the laboratory using *D. sibiricus*, *M. sexta*, *M. brassicae*, and *S. cerealella* eggs. The parasitoid remained viable and efficiently attacked eggs of various lepidopteran hosts. The present study extends the knowledge of susceptible hosts of *T. dendrolimi.* Some of these host species provide high reproduction indices of the parasitoid and could be used for large scale propagation of this biocontrol agent.

## 1. Introduction

Oviphagous hymenopterans of the genus *Trichogramma* (Hymenoptera: Trichogrammatidae) are important endoparasites of insect eggs, showing great potential for pest control of agricultural and forest ecosystems. Over 240 species are described within this genus [1,2,3]. Among those, as many as 230 species have been tested against a wide range of economically important lepidopteran pests, and over 50 species are exploited worldwide as commercial biocontrol agents [2].

The majority of *Trichogramma* species are broadly polyphagous, though their host range and efficiency may vary remarkably among species. Effectiveness against specific insect species is a key feature of a biocontrol tool, since it directly determines the economic effect achieved by usage of a particular species of parasitoid against a target pest species or a species complex. It depends on specificity of the natural enemy and the numbers released [4]. In addition, production cost affects the economic feasibility of pest control using a particular natural enemy, primarily dependent upon organization of the most cost-efficient process of mass rearing of standardized high-quality insects. The ability to parasitize a broad range of host eggs of the *Trichogramma* species made it possible to use the diversity of susceptible hosts for development of mass production technologies. In global practice, the most widely used host species for in vivo reproduction of *Trichogramma* belong to the moth superfamily Pyraloidea (*Duponchelia fovealis* (Zeller, 1847), *Ephestia* (*Anagasta*) *kuehniella* (Zeller, 1879), *Plodia interpunctella* (Hubner, 1813), *Corcyra cephalonica* (Stainton, 1866) etc.) and family Gelechiidae (*Sitotroga cerealella* (Olivier, 1789), *Pectinophora gossypiella* (Saunders, 1844), etc.) [5,6,7,8]. The main advantage of these approaches is the low level of labour costs incurred by insect host rearing [9,10]. However, significant drawbacks for these technologies are known [11,12], including a decrease in the quality characteristics of the produced insect over time. In particular, the proportion of normally developed individuals can decrease [12,13], the sex ratio can change in favour of males [13], and the adult size and fertility can diminish, seemingly linked to the small size of the host eggs [14]. Adaptation to the substitute laboratory artificial host may also develop, decreasing the field activity against the target pest species [1,15]. The usage of host eggs of larger dimensions, which favours production of higher numbers of parasitoids, may help avoiding the negative effects of smaller host eggs [1].

The oviphagous parasitoid *T. dendrolimi* (Matsumura, 1926) is a widespread Eurasian species found in many European and Asian countries, including Netherlands, France, Germany, Poland, Ukraine, Greece, Italy, Turkey, Russia, Iran, Kazakhstan, India, China, Japan, and others [16]. Attempts to introduce it to South America have also been reported [17]. The trophic spectrum of *T. dendrolimi* include over 140 species of lepidopterans belonging to 23 families [16]. This species is widely utilized in biological control programmes. In China, *T. dendrolimi* is released to combat field crop pests *Ostrinia furnacalis* (Guenee, 1824), *O. nubilalis* (Hubner, 1796), *Ectropis grisescens* (Warren, 1894), *Mythimna separata* Walker, 1865, *Mamestra brassicae* (L., 1758), *Helicoverpa armigera* (Hubner, 1808), and *Chilo supressalis* (Walker, 1863); the fruit and berry pest *Samia cynthia ricini* (Drury, 1773)*;* and the forest pests such as *Dendrolimus punctatus* (Walker, 1855) and *Antheraea pernyi* (Guerin-Meneville, 1855) [18,19]. Modern laboratory studies and field trials are aimed at extending fundamental knowledge of the biological properties in a search for promising practical applications of *T. dendrolimi*. As many as 65 relevant scientific publications for the period of 2019–2024 can be found in the Google Scholar bibliographic database.

The success of a biological control programme is dependent on numerous factors, and host specificity of a biocontrol agent is among the key ones. It is expected that the *Trichogramma* species and strains will be effective against the particular host species which they have been isolated from, i.e., the original host [20]. On the other hand, levels of parasitism may be even higher when the parasitoid switches to an alternative host. For example, *T. ostriniae* Pang, Chen, 1974 isolated from *O. nubilalis* parasitized more host eggs and showed higher fecundity, emergence from parasitized eggs and female ratio in *Helicoverpa zea* (Boddie, 1850) (Noctuidae) than in the original host [21]. Though field efficiency of *T. ostriniae* against *O. nubilalis* and *O. furnacalis* is higher compared to *T. dendrolimi*, the latter species is still considered as a promising biocontrol agent against these pests [22,23,24]. It can also be speculated that a parasitoid is able to adapt to particular groups of hosts which are present in the same locality due to adaptation to particular species [25,26].

Intraspecific differences may also be observed in parasitoid performance on different hosts. For example, successful development of *T. ostriniae* was reported on *E. kuhniella* and *S. cereallella* [27,28]. This was not confirmed for another strain of this parasitoid species, though the possibility of its adaptation to these alternative hosts through a series of generations was presumed [21].

For large-scale production needs, *T. dendrolimi* is successfully reared on a number of host insects, including *Antheraea pernyi* (Guerin-Meneville, 1855) [29,30,31], *Corcyra cephalonica* (Stainton, 1866) [19,32], D. *punctatus* [31], and *E. kuehniella* [33]. When selecting a host producer, it is important to consider endogenous and environmental factors that affect the parasitoid in terms of biocontrol effectiveness. These factors may include geographical origin, host adaptation, host egg age, storage and cultivation conditions, etc. [18,34].

In this study, we tried to determine the range of potential hosts of a new strain of *T. dendrolimi* exploiting widespread lepidopteran species, either inhabiting Northern Asia (and potentially exposed to the parasitoid in nature), or available as continuous laboratory cultures (to ensure the ease of cultivation for mass production purposes). Many of those are pests of high economic significance, which highlights the practical importance of the study. We also tested the impact of the host egg size on morphometric parameters of *T. dendrolimi*.

## 2. Materials and Methods

### 2.1. Parasitoid Source and Rearing

The parasitoid was obtained from the parasitized eggs of *D. sibiricus*, collected on the branches of the Siberian larch *Larix sibirica* (Ledeb., 1833) in the pest foci of the Irkutsk region (53°55′30″ N, 105°48′01″ E). Each sample was placed in a 5 mL plastic tube, enclosed with a cotton plug, transported to the laboratory, and kept at +24 °C, relative humidity of 50%, and the photoperiod of 16:8 (day/night) until the emergence of adult parasitoids. Identification of *T. dendrolimi* was based on morphometric characteristics of male individuals using the taxonomic keys [32], supplemented by DNA barcoding using a fragment of the mitochondrial cytochrome oxidase I gene, GenBank accession numbers OR732466; OR732435; OR732455; OR732453 [35].

Newly emerged parasitoid adults were placed in new test tubes (30 mL), and a 50% aqueous solution of honey was applied in a thin strip along the tube wall. For several generations, the parasitoid culture was maintained using fresh eggs of the original host. For this purpose, on the second day after emergence of the parasitoid adults in the test tubes, they were supplied with paper sheets covered with the host eggs, glued by sugar syrup, for 24 h. Then, the sheets were withdrawn and stored as above until emergence of the adults of the next generation. The stock culture of the parasitoid has been maintained over the years of experiments for as many as 30 generations on the eggs of several available laboratory hosts, namely *D. sibiricus*, *Manduca sexta* (L., 1763), *M. brassicae* (L., 1758), and *S. cerealella* (Olivier, 1789) (see below).

### 2.2. Host Source and Rearing

For bioassays, lepidopteran insects were used, available either as temporary cultures based on field-collected samples, or as permanent laboratory cultures. They were chosen for having pest status and/or shared ecological niche with the parasitoid (as potential alternative hosts in nature), or for being model species used for laboratory rearing. A total of 17 species were tested, which can be divided into two groups based on their ecological and geographical association with the parasitoid.

The first group included the species whose geographic range and ecosystem affinity coincide with the collection area of the parasitoid strain under study. Using light traps, the adults of *Chrysoithrum flavomaculata* (Bremer, 1861), *Orgyia antiqua* (L., 1758), *Angerona prunaria* (L., 1758), *Odontopera bidentata* (Clerck, 1759), *Macrothylacia rubi* (L., 1758), *Pseudoips prasinana* (L., 1758), *Deilephila porcellus* (L., 1758), and *Mimas tiliae* (L., 1758) were sampled. Larvae of the original host *D. sibiricus* (Tschetverikov, 1908) were collected on branches of Siberian larch in the Irkutsk region and maintained on the host plant until pupation, followed by adult emergence. We refer to this entire group as “sympatric” in the context of the current study.

The second group represented agricultural and forest pests which are not associated with the geographic area of the parasitoid strain, suggesting absence of ecological interactions with the latter. We name these species “allopatric”. Larvae of *Dendrolimus pini* (L., 1758) were collected by hand from the Scotch pine plantings in the Altai Area. Larvae of *Hyphantria cunea* (Drury, 1773) were collected in Krasnodar Area on mulberry trees growing in the wild. Larvae of *Hydraecia micacea* (Esper, 1789) were collected on an experimental potato field at the territory of All-Russian Institute of Plant Protection (St. Petersburg). The field-collected larvae were maintained on their respective natural host plants until pupation, followed by adult emergence. Pupae of *M. sexta* from a permanent culture were kindly provided by T-Rex Food^®^ (Moscow, Russia) and maintained at room temperature until adult emergence.

Adult specimens captured in nature or emerged under laboratory conditions were kept at room temperature in plastic containers (2 or 4 L), supplied with pieces of cotton wool soaked in sugar syrup for additional feeding and paper sheets for laying eggs. In the case of *M. sexta*, the adults were kept in a nylon mesh cage (flexarium), and eggs were collected from its walls with a brush.

Continuous laboratory cultures of *Galleria mellonella* (L., 1758), *M. brassicae*, and *Ostrinia nubilalis* (Hubner, 1796) were routinely reared at the All-Russian Institute of Plant Protection. Larvae were fed with respective artificial diets, eggs were laid by the adult moths on paper sheets and the pieces of paper containing the eggs were excised. Ready-to-use eggs of *S. cerealella* (Olivier, 1789) from a permanent culture were purchased from Institute of Applied Entomology (St. Petersburg, Russia) (Table 1).

For each tested lepidopteran species, 20 eggs were sampled randomly from eggs of several females. For each egg specimen, radius was measured three times (in different dimensions) to calculate the egg volume [36].

### 2.3. Egg Parasitism Bioassay

Paper sheets with a glue layer were covered with freshly laid host eggs (usually up to 4 h) and placed in test tubes with the *T. dendrolimi* adults. To avoid excessive parasitism that causes egg spoilage [37,38], specific ratios were chosen (Table 2). The exposure period for 1-day-old and 3-day-old adult parasitoids was 12 and 24 hrs, respectively. After exposure, the eggs were transferred to new 5 mL plastic tubes, capped with cotton plugs and stored as above. After 5 days, the host eggs were examined under a stereomicroscope Micromed MC-4-ZOOM (LOMO, St. Petersburg, Russia), and egg parasitism levels were assessed by the characteristic chorion colour change to graphite. To estimate the number of parasitoids emerged from one egg (total individuals per egg), 20 eggs of each assayed host species where parasitism was successful were placed individually in 2 mL plastic tubes and stored as above for parasitoid counting. After adult emergence, the eggs were repeatedly examined. The cases of complete development to adult stage, followed by exit, as well as formation of pupae or adults which perished within the host eggs, were considered “successful parasitism”. The cases of deformed chorion of host eggs, presumably due to damage by the parasitoid ovipositor, as well as parasitoids perished at the larval stage, were considered “non-productive parasitism”. The cases of unaffected host eggs (showing no signs of piercing by the parasitoid ovipositor, the colour left unchanged, or with host larvae hatched) were considered “unsuccessful parasitism”.

### 2.4. Morphometric Measurements

**Figure 1:**
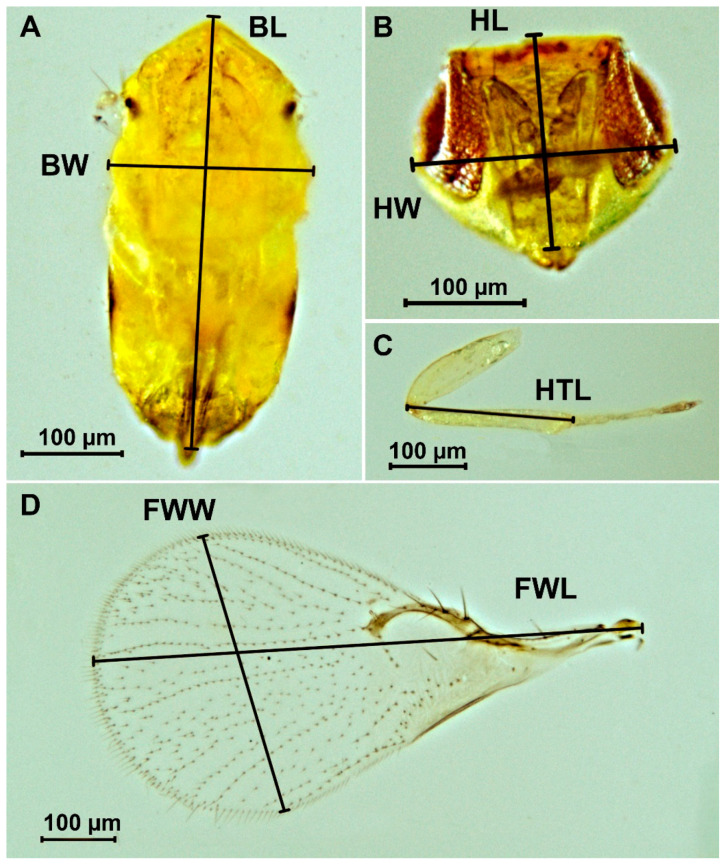
Measurement of morphometric parameters of body (**A**), head (**B**), hind leg (**C**) and front wing (**D**) of the female *Trichogramma dendrolimi*. BL: body length, BW: body width, FWL: forewing length, FWW: forewing width, HL: head length, HTL: hind tibia length, HW: head width [32].

To assess the dimensional variability of *T. dendrolimi*, a total of 20 adult female specimens were chosen from each host species in which the parasitoid development was successful. The preparations were made based on the method of Platner et al. [39]. The body, head, hind leg, and front wing were prepared. Morphometric measurements were performed using a light microscope Micromed 2 (LOMO, St. Petersburg, Russia) and digital camera MAGUS CHD30, supplied with MAGUS View software, version x64, 4.11.24030.20231203 [40]. The following parameters were assessed: body length, body width, head length, head width, hind tibia length, forewing length, and forewing width, as shown in Figure 1.

### 2.5. Data Analysis

All statistical analyses were conducted in R (version 4.4.2) [41]. Fisher’s exact test (package ‘stats’) with Holm-Bonferroni correction compared frequencies of successful parasitism, non-productive parasitism, and unsuccessful parasitism among host species, using the native host *D. sibiricus* as the reference. Comparisons included each host species versus the reference group and geographically associated versus non-associated host groups. For each comparison, odds ratios with 95% confidence intervals and corresponding *p*-values were calculated using 2 × 2 contingency tables [42], with statistical significance determined at adjusted *p*-values < 0.05. Cases with zero cell frequencies were addressed through continuity corrections (adding 0.5 to cells when needed) and exact boundary estimation methods to ensure robust parameter estimation.

For host suitability analysis, we examined three key parameters: (i) number of parasitoid individuals emerging from single host egg clutches, (ii) morphometric measurements of female parasitoids from different host species, and (iii) comparative analysis of host egg clutch size. Parametric comparisons of egg clutch size and morphometric data utilized Welch’s ANOVA followed by Games-Howell post hoc tests (package ‘userfriendlyscience’), selected for their robustness to heteroscedasticity. However, for parasitoid yield per host egg—particularly for groups exhibiting zero variance (*G. mellonella*, *H. micacea*, *O. nubilalis*, *S. cerealella*)—we applied non-parametric Kruskal–Wallis tests with Dunn’s post hoc tests incorporating Holm’s correction for multiple comparisons.

The relationship between host egg size and number of developed adult parasitoids was further investigated using linear regression analysis, with diagnostic plots verifying model assumptions of linearity, homoscedasticity, and normality of residuals.

## 3. Results

### 3.1. Parasitism of the Tested Host Species

Under laboratory conditions, the examined *T. dendrolimi* strain showed sustainable development in the eggs of its original host, the Siberian silk moth, which served as the standard in the bioassays performed. On average, the cases of successful parasitism exceeded 41%, while non-productive and unsuccessful parasitism constituted around 17% and 42%, respectively. Among the 17 species of the tested lepidopterans (including the original host), only three species were not parasitized, namely *D. porcellus*, *M. tiliae*, and *H. cunea*. These eggs showed no visible changes in colour or morphology. Although female parasitoids visited them during the experiment, they did not linger or oviposit.

The other 13 species could serve as alternative hosts for the complete developmental cycle of the parasitoid under laboratory conditions, with successful parasitism cases exceeding at least 10%. The distribution of the successful, non-productive and unsuccessful parasitism cases among these host taxa were not equal. The least parasitized were *A. prunaria* and *O. nubilalis*, with 12% and 16% of successful parasitism, respectively; all other cases were represented by unsuccessful parasitism (i.e., there was no non-productive parasitism). In addition, the low level of successful parasitism, about 15%, was observed in *M. brassicae*, but non-productive parasitism reached 83%. The group with mediocre parasitism parameters consisted of *M. rubi*, *D. pini*, *M. sexta*, *H. micacea*, and *S. cereallella*, with successful parasitism in the range of 40–50% and non-productive—below 20%. Similarly, *O. antiqua* showed 52% of successful parasitism, though non-productive cases reached 46%. *Galleria mellonella* showed moderately higher successful parasitism frequency—56%. As for the species showing the highest levels of successful parasitism, those included *O. bidentata* (87%), *P. prasinana* (79%), and *C. flavomaculata* (86%) (Table 3).

The outcome is presented as successful parasitism (SP), non-productive parasitism (PP), and unsuccessful parasitism (UP)

**Figure 2:**
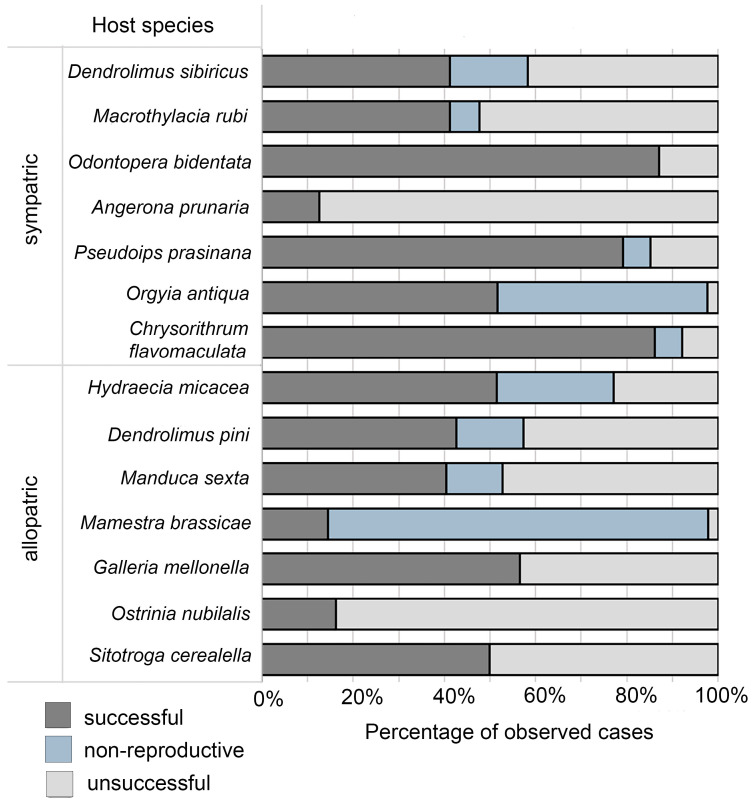
Indices of *Trichogramma dendrolimi* parasitism of eggs of lepidopteran insect hosts under experimental conditions in sympatric and allopatric hosts (see Materials and Methods, Section 2.2). The proportions of cases of successful parasitism (SP), non-productive parasitism (PP), and unsuccessful parasitism (UP).

Combining successful and non-productive parasitism as the single index of biological efficacy (as both states correspond to the killing of the target pests) provided some corrections to delineation of these groups. Namely, the least affected hosts included only *A. prunaria* and *O. nubilalis*, while *M. brassicae*, *O. antiqua*, and *H. micacea* joined the most affected species (Figure 2).

The analysis revealed pronounced geographic specificity in parasitoid-host interactions: the allopatric group of host species showed a 2.2-fold lower probability of successful parasitism and a 1.8-fold higher frequency of non-productive parasitism as compared to the sympatric hosts.

### 3.2. Dependence of the Number of Parasitoids on the Size of the Host Egg

**Figure 3:**
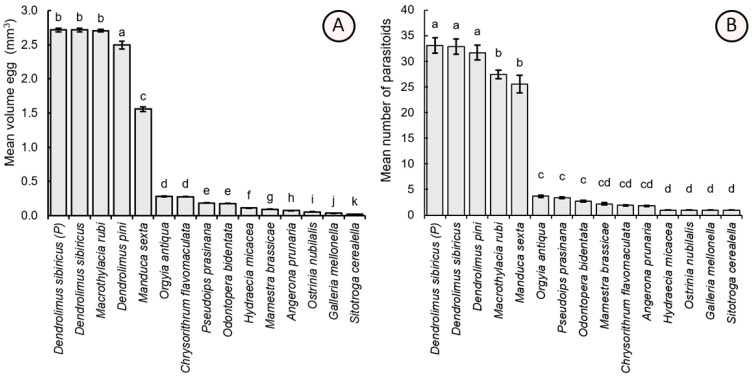
Mean (±SE) egg volume (mm^3^) of the tested host species (**A**) and the mean number of *Trichogramma dendrolimi* adults hatched from each host egg (**B**). Significant differences in the columns are indicated by different letters: *p* < 0.05; Games-Howell test (**A**), Dunn’s test (**B**). P—parental generation, i.e., the field-sampled insects of *D. sibiricus* were exploited to obtain the eggs for the bioassays, as opposed to the laboratory-cultivated culture of this host.

Egg size varied significantly among host species (F_14,285_ = 2884.80, *p* < 0.001). The largest eggs were found in *D. sibiricus*, followed by other lasiocampids (tent caterpillars) such as *D. pini* and *M. rubi*, which had slightly smaller eggs. *Manduca sexta* eggs were about twice as smaller than those of lasiocampids but still significantly larger than most other tested hosts, including *O. antiqua*, *P. prasinana*, *O. bidentata*, *M. brassicae*, *C. flavomaculata*, *A. prunaria*, *H. micacea*, *O. nubilalis*, *G. mellonella*, and *S. cerealella*. Among these, *S. cerealella* had the smallest eggs (Figure 3A).

The number of adult *Trichogramma* individuals hatched from the eggs of different host species also differed significantly (χ^2^ = 255.28, df = 14, *p* < 0.001). The highest number of the parasitoids hatched from the large eggs of parental *D. sibiricus* (total individuals per egg = 33.10 ± 1.54), laboratory-cultivated *D. sibiricus* (32.90 ± 1.48), and *D. pini* (31.70 ± 1.45). A mean of 27.40 ± 0.85 individuals per egg hatched from *M. rubi*, and a similar value was observed for *M. sexta* (25.55 ± 1.70). Small-sized eggs produced significantly fewer fully developed adults in *O. antiqua* (3.70 ± 0.21), *P. prasinana* (3.40 ± 0.18), *O. bidentata* (2.70), *M. brassicae* (2.20 ± 0.20), *C. flavomaculata* (1.90 ± 0.07), *A. prunaria* (1.80 ± 0.09), *H. micacea* (1.00), *O. nubilalis* (1.00), *G. mellonella* (1.00), and *S. cerealella* (1.00) (Figure 3B).

Linear regression analysis of the dependence of the number of developed adult *Trichogramma* individuals per host egg (TIE) on the egg size (V) of the tested host species revealed a positive significant correction: TIE = 0.74 × V + 0.98 (R^2^_adjusted_ = 0.672, F_14,285_ = 696.7, *p* < 0.001).

### 3.3. Morphometric Parameters of the Parasitoid

**Figure 4:**
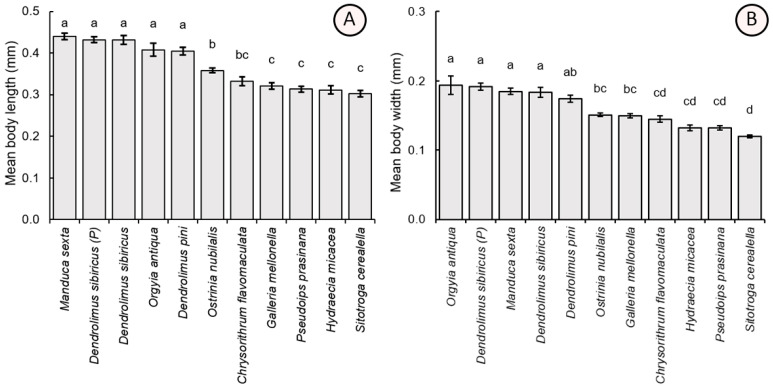
Comparison of the mean values (±SE) of the body length (**A**) and width (**B**) of *Trichogramma dendrolimi* reared in eggs of different host species. Significant differences are indicated by different letters (*p* < 0.05, Games-Howell test). P—see Figure 3.

Morphometric parameters of parasitoid females reared from the eggs of the tested lepidopteran species, including the parasitoids obtained from the natural population, showed significant differences. In particular, there was a significant difference in the parameters of body length (F_10,209_ = 40.17, *p* < 0.001) and width (F_10,209_ = 39.92, *p* < 0.001). The largest specimens were obtained from eggs of laboratory-cultivated *D. sibiricus* (body length = 0.432 ± 0.049 mm, body weight = 0.184 ± 0.032 mm), parental *D. sibiricus* (0.432 ± 0.036 mm, 0.192 ± 0.024 mm), *M. sexta* (0.440 ± 0.036 mm, 0.185 ± 0.020 mm), *D. pini* (0.405 ± 0.043 mm, 0.174 ± 0.022 mm), and *O. antiqua* (0.408 ± 0.070 mm, 0.194 ± 0.061 mm). Morphometric values of *T. dendrolimi* adults reared from hosts possessing the larger eggs were significantly higher as compared to those obtained from the hosts with the smaller eggs: *O. nubilalis* (0.358 ± 0.027 mm, 0.151 ± 0.013 mm), *G. mellonella* (0.321 ± 0.035 mm, 0.149 ± 0.015 mm), *C. flavomaculata* (0.332 ± 0.047 mm, 0.145 ± 0.020 mm), *H. micacea* (0.311 ± 0.046 mm, 0.132 ± 0.018 mm), *P. prasinana* (0.313 ± 0.030 mm, 0.132 ± 0.014 mm), and *S. cerealella* (0.302 ± 0.034 mm, 0.120 ± 0.009 mm) (Figure 4).

**Figure 5:**
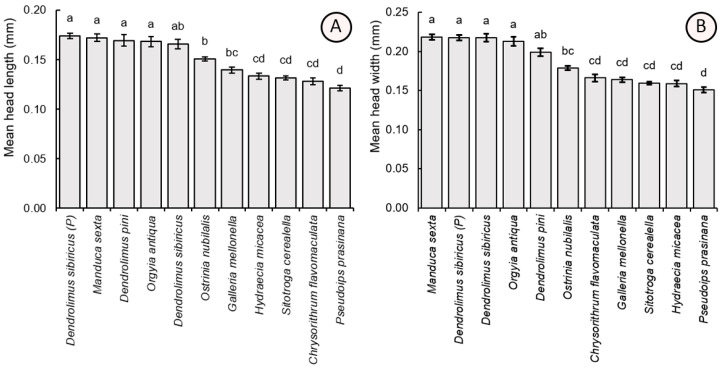
Comparison of the mean values (±SE) of the head length (**A**) and width (**B**) of *Trichogramma dendrolimi* reared in eggs of different host species. Significant differences are indicated by different letters (*p* < 0.05, Games-Howell test). P—see Figure 3.

Parasitoid individuals also differed significantly in the parameters of head length (F_10,209_ = 38.12, *p* < 0.001) and width (F_10,209_ = 55.15, *p* < 0.001). Mean (±SE) head capsule dimensions were comparable for wasps reared from hosts with large eggs: parental *D. sibiricus* (head length = 0.174 ± 0.013 mm, head width = 0.218 ± 0.015 mm), *M. sexta* (0.172 ± 0.016 mm, 0.219 ± 0.016 mm), laboratory-cultivated *D. sibiricus* (0.165 ± 0.022 mm, 0.217 ± 0.022 mm), *D. pini* (0.169 ± 0.025 mm, 0.199 ± 0.022 mm), and *O. antiqua* (0.168 ± 0.022 mm, 0.213 ± 0.026 mm). These values were significantly higher as compared to the individuals obtained from *O. nubilalis* (0.150 ± 0.009 mm, 0.179 ± 0.011 mm), *G. mellonella* (0.139 ± 0.040 mm, 0.150 ± 0.013 mm), *C. flavomaculata* (0.128 ± 0.015 mm, 0.166 ± 0.020 mm), *H. micacea* (0.133 ± 0.013 mm, 0.159 ± 0.016 mm), *S. cerealella* (0.131 ± 0.009 mm, 0.160 ± 0.009 mm), and *P. prasinana* (0.121 ± 0.011 mm, 0.151 ± 0.015 mm) (Figure 5).

**Figure 6:**
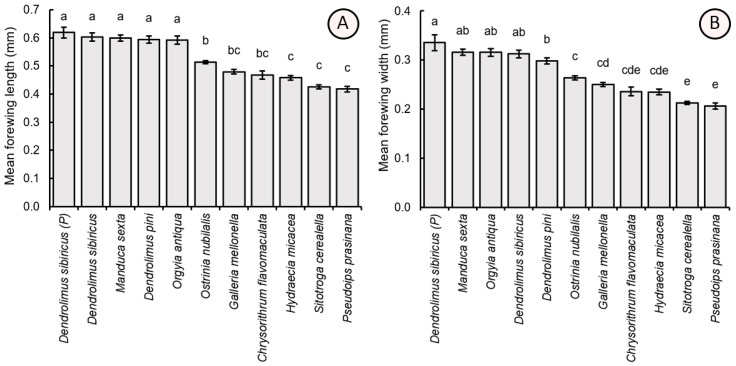
Comparison of the average value (±SE) of the length (**A**) and width (**B**) of the forewing of female *T. dendrolimi* reared from the eggs of different host species. Significant differences in the columns are indicated by different letters (*p* < 0.05, Games-Howell test). P—see Figure 3.

The length and width of the forewing of the parasitoid specimens also showed significant differences depending on the host egg size species (F_10,209_ = 45.30, *p* < 0.001 and F_10,209_ = 52.81, *p* < 0.001, respectively). The individuals possessing larger bodies and heads also had larger forewings, including those reared from parental *D. sibiricus* (forewing length = 0.618 ± 0.084 mm, forewing width = 0.335 ± 0.072 mm), *M. sexta* (0.600 ± 0.050 mm, 0.316 ± 0.027 mm), *D. sibiricus* (0.603 ± 0.067 mm, 0.312 ± 0.035 mm), *D. pini* (0.594 ± 0.056 mm, 0.298 ± 0.028 mm), and *O. antiqua* (0.591 ± 0.065 mm, 0.315 ± 0.036 mm). These values significantly exceeded the forewing dimensions of the specimens (which in turn displayed smaller bodies and heads) obtained from *O. nubilalis* (forewing width = 0.513 ± 0.027 mm, forewing width = 0.264 ± 0.017 mm), *G. mellonella* (0.479 ± 0.037 mm, 0.250 ± 0.020 mm), *C. flavomaculata* (0.467 ± 0.067 mm, 0.236 ± 0.038 mm), *H. micacea* (0.458 ± 0.037 mm, 0.235 ± 0.024 mm), *S. cerealella* (0.425 ± 0.031 mm, 0.213 ± 0.014 mm), and *P. prasinana* (0.418 ± 0.046 mm, 0.206 ± 0.027 mm) (Figure 6).

**Figure 7:**
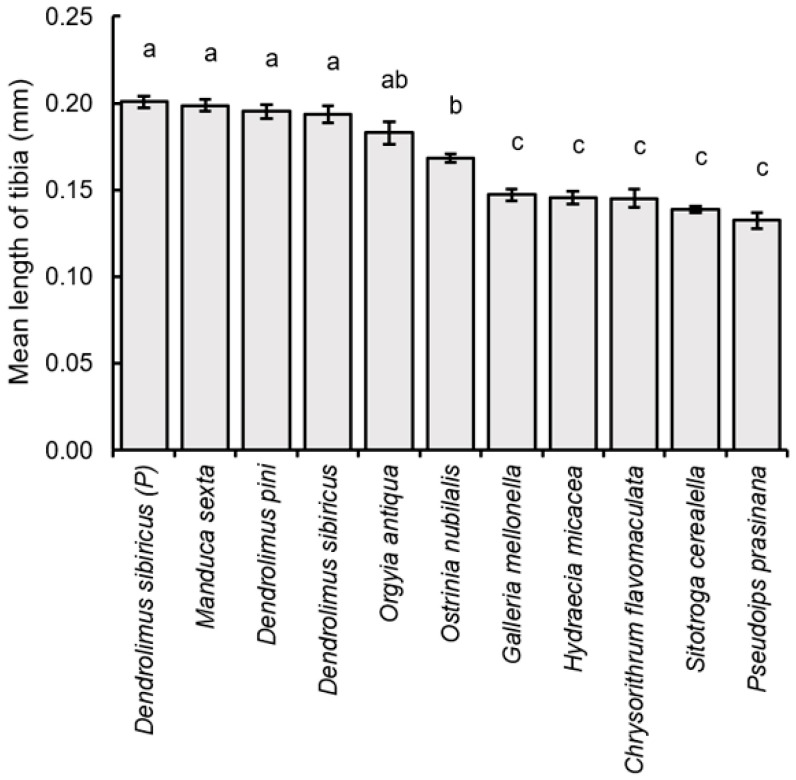
Comparison of the average value (±SE) of the tibia length of the hind leg *of Trichogramma dendrolimi* females, reared in eggs of different host species. Significant differences in the columns are indicated by different letters (*p* < 0.05, Games-Howell test). P—see Figure 3.

Finally, the larger parasitoid specimens also had longer hind tibia (F_10,209_ = 56.07, *p* < 0.001), including those obtained from parental *D. sibiricus* (hind tibia length = 0.201 ± 0.015 mm), *M. sexta* (0.199 ± 0.015 mm), *D. sibiricus* (0.194 ± 0.023 mm), *D. pini* (0.195 ± 0.018 mm), and *O. antiqua* (0.183 ± 0.029 mm). These values were not statistically different between each other, but significantly exceeded those obtained from *O. nubilalis* (0.168 ± 0.012 mm), *G. mellonella* (0.147 ± 0.015 mm), *C. flavomaculata* (0.145 ± 0.023 mm), *H.micacea* (0.146 ± 0.016 mm), *S. cerealella* (0.139 ± 0.009 mm), and *P. prasinana* (0.132 ± 0.020 mm), with the only exception of *O. antiqua* and *O. nubilalis*, which were not significantly different between each other (Figure 7).

## 4. Discussion

Information on the spectrum of potential host species, including those belonging to the local fauna, as well as the substitute laboratory hosts, is essential to ensure effective development and application of *Trichogramma* biocontrol agents. In addition, knowledge and consideration of biological features that limit the population state of a culture are also important factors that determine the effectiveness of the biological control agents [18,22,34].

Under natural conditions in Siberia, the Siberian silk moth *D. sibiricus* is the most serious defoliator of coniferous forests [43,44]. A fairly wide complex of natural enemies is known for the Siberian silk moth, including oviphagous parasitoids, such as *Telenomus tetratomus* (Kieffer, 1909), *Ooencyrtus pinicolus* (Matsumura, 1926), and *T. dendrolimi* [35], contributing to its population dynamics during pest outbreaks [35,45,46,47,48]. Meanwhile, in the timeframe between the outbreaks, the Siberian silk moth develops mainly in a two-year generation mode, including two consequent hibernation periods during the two respective winter seasons [45]. This also prevents the natural enemies, which parasitize the egg stage, from maintaining the stable regulatory effect on the host, allowing its populations to liberate from the pressure of parasitoids [35].

Nevertheless, the egg parasitoids continue to persist in the forest ecosystems on annual basis, regardless of the silk moth generation cycles. In particular, *T. dendrolimi* is a polyvoltine species and can have several generations per season, which evidently cannot be synchronized with *D. sibiricus* oviposition [4]. Hence, other lepidopteran hosts, inhabiting the same geographical area, should serve as additional hosts of the parasitoids to ensure their population persistence in a given locality. Testing these insects as hosts of the parasitoid extends our understanding of its biology. The utilization of hundreds of eggs in numerous repetitions, assayed for the majority of lepidopteran species under present study, allowed us to generate robust conclusions concerning estimated host range of *T. dendrolimi.* A limited number of eggs was obtained for two species only, namely *M. tiliae* and *D. porcellus.* Yet, we suppose that the sample sizes used in these cases are sufficient for the purposes of the present study to make the appropriate conclusions (see below). And though all the tests were performed under laboratory conditions, it can be expected that the parasitoid will act against the respective hosts in nature, as repeatedly demonstrated in numerous studies [1,18,27].

Most *Trichogramma* species are reported to parasitize a wide range of hosts from different lepidopteran families, and *T. dendrolimi* is no exception [16]. The present study confirmed its ability to attack the host species previously known to be susceptible to this parasitoid, including the original lasiocampid host *D. sibiricus*, its congener *D. pini*, erebid *O. antiqua*, gelechiid *S. cerealella*, pyralids *G. mellonella* and *O. nubilalis*, and noctuid *M. brassicae*. Furthermore, the suitability of lasiocampid *M. rubi*, erebids *C. flavomaculata*, geometrids *A. prunaria* and *O. bidentata*, and nolid *P. prasinana* for *T. dendrolimi* egg parasitism is displayed for the first time. These species, as well as *O. antiqua* and other hosts beyond the current examination, inhabit similar ecological conditions and their geographic area partly overlaps with that of *D. sibiricus.* It is therefore logical to assume that these species can serve as additional hosts in nature, especially during periods when the Siberian silk moth eggs are absent.

Other hosts for which *T. dendrolimi* parasitism is proved for the first time are the noctuid *H. micacea* and the sphingid *M. sexta.* The former is a polyphagous pest which occasionally damages different crops in Europe [49] and was also introduced to Canada [50]. Recent reports highlight its damage to hop plantations in Europe [51]. The detection of *T. dendrolimi* parasitism in this noctuid is consistent with the broad spectrum of its already known noctuid hosts [16]. Comparatively high levels of both successful and non-productive parasitism allow consideration of *T. dendrolimi* as an effective biocontrol agent against this pest.

Interestingly, among the three sphingid species assayed, only *M. sexta* was parasitized by the parasitoid. This extends the known range of sphingid hosts of *T. dendrolimi*, since the only previously reported host from this family was *Clanis bilineata* [16]. The importance of *M. sexta* is augmented by the fact that it easily reproduces in captivity and is exploited for biotechnological applications [52]. It may represent a reasonable alternative to other sphingids that are mass-propagated for industrial purposes, such as *C. bilineata* [53]. Mass production of *M. sexta* has been studied for a long time [54,55,56]. Large host body dimensions and sizeable eggs facilitate maintenance of the parasitoid’s body measurements corresponding to that obtained in the original host. Meanwhile, the observed body size shrinking of *T. dendrolimi* in eggs of smaller hosts is a drawback, as it negatively affects the fecundity, longevity, sex ratio, and parasitism levels of the biocontrol agent [14,20]. For example, a natural strain of *Trichogramma* (also designated as *T. dendrolimi*, though with no background information provided on identification protocol) was isolated from a field population of *M. brassicae* and passaged on a substitute laboratory host, the minute pyralid *E. kuehniella*. As a result, the body proportions decreased and the number of eggs laid by the parasitoid was two-fold lower compared to the original host. Importantly, even after 12 generations maintained on *E. kuehniella*, *T. dendrolimi* preferred the eggs of the former host over the latter one [57]. This observation raises the possibility of high efficiency against the target pests even after long-term cultivation of the egg parasitoid in alternative hosts. Propagation in host eggs of larger size (as in *M. sexta*) may also contribute to increased efficiency due to enhanced fitness of the parasitoid showing increased dimensional characteristics. Nevertheless, the host switching ability of *T. dendrolimi* should be checked after continuous propagation in eggs of alternative hosts. Testing host specificity of different strains of *T. dendrolimi* species would be helpful for evaluating possible intraspecific differences.

## 5. Conclusions

The new *T. dendrolimi* isolate displayed ability to infest a variety of hosts among Lepidoptera from several families, extending the known host range of this species. The susceptible species represent either potential natural hosts, substitute laboratory hosts, or target pests to be controlled. Among the susceptible hosts, those with the larger eggs are the most promising candidates for mass rearing of the biocontrol agent. The successful reproduction of the new isolate for 30 generations allows considering the established culture as a stable laboratory line.

## Figures and Tables

**Table 1:** Sampling sites of lepidopteran insects in nature and origin of laboratory cultures.

Host Species	Sampling Site/Culture Origin	Geographic Coordinates
*Dendrolimus sibiricus*	Irkutsk Province, Kachugskiy district	54°0′34.18″ N, 106°8′44.91″ E
*Macrothylacia rubi*	Krasnoyarsk Area, Yemelyanovskiy District	56°1′43.20″ N, 92°38′38.94″ E
*Dendrolimus pini*	Altai Area, Klyuchevsky District	52°15′11.31″ N, 78°57′49.36″ E
*Deilephila porcellus*	Krasnoyarsk Area, Yemelyanovsky District	55°58′45.58″ N, 92°38′55.40″ E
*Mimas tiliae*	Krasnoyarsk Area, Yemelyanovsky District	55°58′45.58″ N, 92°38′55.40″ E
*Odontopera bidentata*	Irkutsk Province, Kachugskiy District	53°58′17.53″ N, 105°54′24.50″ E
*Angerona prunaria*	Krasnoyarsk Area, Yemelyanovsky District	55°58′45.58″ N, 92°38′55.40″ E
*Pseudoips prasinana*	Krasnoyarsk Area, Yemelyanovsky District	56°1′43.20″ N, 92°38′38.94″ E
*Orgyia antiqua*	Irkutsk Province, Kachugskiy District	54°0′34.18″ N, 106°8′44.91″ E
*Chrysorithrum flavomaculata*	Irkutsk Province, Kachugskiy District	54°0′34.18″ N, 106°8′44.91″ E
*Hyphantria cunea*	Krasnodar Area, Slavyanskiy District	45°40′13.18″ N, 37°47′42.27″ E
*Hydraecia micacea*	St. Petersburg, Pushkinskiy District	59°44′22.82″ N, 30°25′34.83″ E
*Galleria mellonella*	All-Russian Institute of Plant Protection, St. Petersburg	
*Mamestra brassicae*	All-Russian Institute of Plant Protection, St. Petersburg	-
*Manduca sexta*	T-Rex Foods, Moscow	-
*Ostrinia nubilalis*	All-Russian Institute of Plant Protection, St. Petersburg	
*Sitotroga cerealella*	Institute of Applied Entomolgy, St. Petersburg	

**Table 2:** Number of host eggs provided per *Trichogramma dendrolimi* female depending upon the egg size.

Host Species		Number of Eggs Tested	Number of Eggs per Parasitoid Female	Number of Sheets with Eggs	Egg Size Ranking *
Lasiocampidae	*Dendrolimus sibiricus*	357	1–2	35	Large
	*Macrothylacia rubi*	378	1–2	35	Large
	*Dendrolimus pini*	502	1–2	40	Large
Sphingidae	*Deilephila porcellus*	20	2–3	4	Medium
	*Manduca sexta*	425	2–3	35	Medium
	*Mimas tiliae*	56	2–3	6	Medium
Geometridae	*Odontopera bidentata*	340	15–20	20	Small
	*Angerona prunaria*	320	15–20	20	Small
Nolidae	*Pseudoips prasinana*	318	15–20	18	Small
Erebidae	*Orgyia antiqua*	420	8–10	20	Small
	*Chrysorithrum flavomaculata*	485	8–10	20	Small
	*Hyphantria cunea*	203	15–20	10	Small
Noctuidae	*Mamestra brassicae*	449	15–20	20	Small
	*Hydraecia micacea*	350	15–20	10	Small
Pyralidae	*Galleria mellonella*	426	15–20	20	Small
Crambidae	*Ostrinia nubilalis*	370	15–20	20	Small
Gelechiidae	*Sitotroga cerealella*	495	15–20	20	Small

* Egg size ranked as small (volume below 1 mm^3^), medium (1–2 mm^3^), and large (over 2 mm^3^).

**Table 3:** Parasitism success variation across host species and geographical groups: statistical comparisons with reference categories.

Host	Outcome	Odds Ratios	95% Confidence Intervals	Significance
Host species compared to the original host				
*Macrothylacia rubi*	SP	1.00	0.74–1.36	*p* > 0.05
	NP	0.33	0.19–0.55	*p* < 0.001
	UP	1.53	1.13–2.08	*p* > 0.05
*Dendrolimus pini*	SP	1.06	0.80–1.41	*p* > 0.05
	NP	0.84	0.57–1.24	*p* > 0.05
	UP	1.04	0.78–1.34	*p* > 0.05
*Manduca sexta*	SP	0.97	0.72–1.31	*p* > 0.05
	NP	0.67	0.44–1.03	*p* > 0.05
	UP	1.25	0.93–1.68	*p* > 0.05
*Odontopera bidentata*	SP	9.57	6.48–14.38	*p* < 0.001
	NP	-		
	UP	0.21	0.14–0.31	*p* < 0.001
*Angerona prunaria*	SP	0.21	0.13–0.31	*p* < 0.001
	NP	-		
	UP	9.74	6.51–14.83	*p* < 0.001
*Pseudoips prasinana*	SP	5.44	3.82–7.82	*p* < 0.001
	NP	0.31	0.17–0.54	*p* < 0.001
	UP	0.24	0.16–0.36	*p* < 0.001
*Orgyia antiqua*	SP	1.53	1.14–2.05	*p* > 0.05
	NP	4.11	2.92–5.87	*p* < 0.001
	UP	0.03	0.02–0.06	*p* < 0.001
*Chrysorithrum flavomaculata*	SP	8.88	6.31–12.63	*p* < 0.001
	NP	0.31	0.19–0.50	*p* < 0.001
	UP	0.12	0.08–0.18	*p* < 0.001
*Mamestra brassicae*	SP	0.24	0.17–0.34	*p* < 0.001
	NP	24.04	16.43–35.69	*p* < 0.001
	UP	0.03	0.01–0.06	*p* < 0.001
*Hydraecia micacea*	SP	1.51	1.11–2.06	*p* > 0.05
	NP	1.68	1.15–2.47	*p* > 0.05
	UP	0.41	0.29–0.58	*p* < 0.001
*Galleria mellonella*	SP	1.86	1.39–2.50	*p* < 0.001
	NP	-		
	UP	1.07	0.80–1.44	*p* > 0.05
*Ostrinia nubilalis*	SP	0.28	0.19–0.40	*p* < 0.001
	NP	-		
	UP	7.19	5.03–10.39	*p* < 0.001
*Sitotroga cerealella*	SP	1.42	1.07–1.89	*p* > 0.05
	NP	-		
	UP	1.40	1.06–1.86	*p* > 0.05
Allopatric hosts compared to sympatric hosts	SP	0.46	0.41–0.51	*p* < 0.001
	NP	1.81	1.55–2.12	*p* < 0.001
	UP	0.58	0.52–0.66	*p* < 0.001

## Data Availability

The raw data supporting the conclusions of this article will be made available by the authors on request.
